# The Fees of Practitioner and Consultant

**Published:** 1920-04-24

**Authors:** 


### TIED HOUSES IN SURGICAL PRACTICE.

### THE FEES OF PRACTITIONER AND CONSULTANT.

**The outspoken essay from a contributor on the subject of "dichotomy" in our last issue ("The Hospital," April 17, 1920, p. 61) has evoked the subjoined expressions of opinion from acknowledged leaders of the medical profession. As will be seen, they take much the same objections to secret fee-splitting as were advanced editorially at the end of the article in question; but they do not deal with one of the suggestions put forward by our correspondent—namely, the practicability of open and acknowledged "dichotomy" as opposed to the secret and possibly corrupt form. Nor do they attempt to view the matter—it is hardly likely that they could—through the eyes of a man who, like the surgeon mentioned by our correspondent, has lost the greater part of his livelihood owing to his war service, and is threatened (through the action of others) with even more complete ruination if he does not consent to a proposal for the clandestine splitting of fees.**

### Sir T. Clifford Allbutt, K.C.B.,

***Regius Professor of Physic at Cambridge.***

I am very sorry to hear from you that the dishonourable practice of demanding on behalf of the family practitioner some fraction of the fee of the consultant may still persist. I had hoped that the whisperings of such traffic had faded away. Personally, this kind of demand was made to me once only, when the family attendant suggested that I should add five guineas to my fee and hand it over to him; seeing, as he remarked, that the patient was a very wealthy man. I told him that if he considered his services might fairly be so valued that I would do as he desired, and that in mentioning my own fee I could add that my colleague thought he ought to have five guineas for his part in the consultation. My colleague hastily replied: "Oh! that wouldn't do at all"; and so the matter dropped. That behind the patient's back blackmail should be levied either upon him (and this is what it would come to), or upon the consultant, is so glaringly corrupt a proposal that I trust there are very few members of our profession who would stoop to look at it. I presume that a barrister who shared his fee with the solicitor would be promptly disbarred? Indeed, I suppose that this transaction, as now alleged to occur occasionally in our own profession, would come under the Corrupt Practices Act. That a rich man may be robbed if a poor one be spared is, I imagine, a tradition from the days of "gentlemanly" brigands.

### Sir George Makins, G.C.M.G., C.B.,

***President Royal College of Surgeons.***

In reply to the request that I would express an opinion on the so-called practice of "dichotomy," I beg unhesitatingly and absolutely to condemn it as unworthy of the medical profession. In your articles in the issue of April 17, certain objections which cannot be refuted are set forth. Your old and valued correspondent stated that the practice "is no new thing; it has already become, we believe, an established custom in some parts of the world, and evidence is not wanting that it is taking root in our own soil." May I point out with regard to this statement that in America it is required as one of the conditions of becoming a Fellow of the College of Surgeons, that the following declaration should be signed. "I hereby promise upon my honour as a gentleman that I will not, so long as I am a Fellow of the American College of Surgeons, practice division of fees in any form; neither by collecting fees for others referring patients to me; nor by permitting them to collect my fees for me; nor will I make joint fees with physicians or surgeons referring patients to me for operation or consultation; neither will I in any way, either directly or indirectly, compensate any one referring patients to me; nor will I utilise any man as an assistant as a subterfuge for this purpose."

As direct evidence of the opinion held of the practice of dichotomy in a soil in which it has to some extent taken root, I think that this declaration should be not without influence on any who may be inclined to view it with leniency on this side of the Atlantic.

### Sir W. Watson Cheyne, Bart., K.C.M.G.

I am afraid I cannot write you a communication on the matter, but I have always felt that the system you speak of was most objectionable. It introduces possibilities which should be avoided, and it is always most satisfactory that the patient should know exactly what he is paying to each person who attends him. The surgeon may, in the case where a patient is not well off, so temper his fee that the patient may still be able to pay the practitioner a suitable remuneration, but I don't think that ought to be done by bargaining, and the patient should be made aware that the fees have been reduced to suit his means.

### Col. Rushton Parker, F.R.C.S., R.A.M.C.T.,

***Professor of Surgery, Liverpool University.***

I have very little experience of dichotomy, and none of tied houses, in surgical practice, but I hold to the view expressed in your editorial remarks on April 17. My own practice and opinions are not inclined to quackery, which is contrary to such notions as I have of self-respect. Nor do I approve of "touting" in any form, and only care to see such patients as wish to see *me*. But I well know that arts of that kind are the chief recommendation on which some persons are able to lean.

Our profession has always contained numerous quacks, of many sorts, and I have long entertained the opinion that no power on earth can stop them.

**Tied Houses in Surgical Practice—(*continued*).**

Those who have the disposition to be quacks will always find the opportunity, and I think many of them make a living which is "very hardly earned." I would not go so far as to say that they deserve all they can make, but personally I would rather keep a beef-and-ham-shop, and have never felt it necessary to resort to quackery. Having studied and practised surgery as an art, I have had no need to manœuvre as an impostor. It is always true that "populus vult decipi," but as a general rule I leave the deception to others.

### Mr. James Swain, C.B., C.B.E., F.R.C.S.

***Professor of Surgery, University of Bristol.***

I AGREE with the main principles embodied in your editorial comment in "Tied Houses in Surgical Practice." Dichotomy is undesirable in the interests of the public and the profession. No layman would like to think that his health, and possibly his life, may be imperilled by an unsuitable consultant who is called in because of his willingness to share fees.

Moreover, it is degrading to practitioner and consultant that the latter should be called in because of a financial agreement, rather than because of the possession of special knowledge.

Patient and medical attendant should not be hampered in the choice of a consultant, and no secret commission should be allowed.

### Sir W. Thorburn, K.B.E., C.B.,

***Professor of Clinical Surgery, Manchester University.***

REPLYING to yours of 16th inst., I think that your Editorial comment upon "Tied Houses in Surgical Practice" quite meets the case. I am most strongly opposed to anything of the nature of what is called "dichotomy," and it appears to me that the usual relations of friendship between general practitioners and consultants fully meet all that is required in the way of co-operation without introducing any bond which is obviously susceptible not only of abuse, but of the suspicion of abuse. From the point of view of the patient, will he knowingly accept the practitioner's selection of a surgeon who pays the latter a commission? If he does not know, is he not deceived? From the practitioner's point of view, does he wish to add to his income by such commissions? From the surgeon's point of view, does he wish to establish a reputation by a method which approaches the bribery which is illegal in business?

